# The Sanitary Service

**Published:** 1898-04-16

**Authors:** 


### The Hospital

A Journal of

The Medical Sciences and Hospital Administration.

Vol. XXIV.—No. 603.] Edited by Sir Henry Burdett, K.C.B., and Solomon C. Smith, M.D., M.R.C.P. [April 16, 1898.

### The Sanitary Service.

The present condition of sanitary law, like everything else in this country, is the result of a slow process of evolution. It does not seem in accordance with the genius of the English people to elaborate *de novo* some new social machinery to carry out some new social want. Rather do we tend to utilise for new necessities any such old machinery as may lie most conveniently to hand, and thus it happened that the sanitary administration of the country had its first origin as an outgrowth from the Poor Law. During the early years of the administration of the Poor Law, the guardians were driven by the circumstances in which they were placed to undertake public health duties, for which at that time no provision was made. They found that the poverty, to deal with which they were appointed, was being increased on every side by fevers and by sickness of all sorts, which were directly attributable to insanitary conditions, and they felt impelled, as a mere matter of economy, to incur charges for preventing the evils which led to so much pauperism. Thus our first, and for a time our only sanitary service consisted of the Poor Law guardians and their medical officers. From this beginning our present sanitary administration has, step by step, developed. Under Local Acts, such as that procured by Liverpool in 1846 (by which Dr. Duncan, the first medical officer of health in the United Kingdom, was appointed); by the Town Improvement Clauses Act in 1847; by the Public Health Act in 1848, by which local boards of health were provided; by the Acts of 1855, of 1872, and finally by the Acts of 1875 for the provinces and 1891 for London districts, and by the Local Government Act of 1888 for counties, the existing sanitary service has been established.

It is to be observed, however, that although the tendency has been, all along, to combine sanitary authorities, to create large sanitary districts, and to introduce the element of county supervision, still, so far as the rural population is concerned, the old association with the Poor Law remains, the guardians being, in fact, the sanitary authority, while even in London the parish, which was originally the unit of the Poor Law, still remains the unit of sanitary administration.

We thus find that the medical department of the sanitary service is in the hands of an infinite number of medical officers for small districts who are the servants of little sanitary authorities, and, although it often happens that one man takes charge of a combined district, he is none the less the servant of each separate board. It is noteworthy, however, that just to the extent to which a medical officer is the servant of his board, to that extent is he the less efficient as an official. For the duties of a medical officer of health by no means consist in doing what he is told. His duty is to advise and to compel, and in no small number of instances he is in this position—that if he does his duty he has to put the law in action against his own paymaster, by whom at any time he may be dismissed from office. For long it has been felt that this is a bad system. The appointment, however, of medical officers to our county councils seems to open the way for a still further step in the evolution of our sanitary service. There can be no doubt that it is best for a district that the medical officer should be independent of his board. At the same time these authorities, not unnaturally, hesitate to make their medical officers entirely independent, and thus to set up kings over themselves whose yoke might not be easy. But if the district medical officers were to be recognised as county officers appointed by the county council; if, in fact, so far as its medical service were concerned, the whole county were administered in unison, and if each district had resident within it, as an advisor, a local medical officer appointed by and constantly in touch with the central body, not only should we find sanitary administration relieved from local influences which are not always good, but we might fairly hope to see far more uniformity in the sanitary condition of different districts.

It is now ten years since the following resolution was passed by the Society of Medical Officers of Health: "That the interests of the public, in regard to efficiency and economy, would be best served by the appointment of the medical officer of health being entirely vested in the county councils." Unfortunately, when the Local Government Bill came before Parliament, there was no opportunity for discussing this proposal, which had however been put in the form of an amendment; but we cannot help feeling that the time has come when it ought to receive the most serious and careful consideration.

It is clear, and especially so in London, that the isolated and inco-ordinate action of adjoining sanitary authorities which prevails at present is most damaging to the public health.

